# Supplementary material for: Radiation dose-response (a Bayesian model) in the radiotherapy of the localized prostatic adenocarcinoma: the reliability of PSA slope changes as a response surrogate endpoint
Source: PeerJ. 2019 Jul 1;7:e7172. doi: 10.7717/peerj.7172 (PMC6610535; doi:10.7717/peerj.7172)
Supplement: Supplemental Information 4 — The dataset comes from a scientific research project approved in Mazandaran University of Medical sciences and was collected in the cancer institute of Tehran, Iran. [file peerj-07-7172-s004.docx]

**Dataset description**

The dataset comes from a scientific research project approved in Mazandaran University of Medical sciences and was collected in the cancer institute of Tehran, Iran. There are two table files: ProstateCancerData.csv and an additional .xls EXCEL file contains the Trajectory PSA values “PSArepeatedmeasurements”.

**1- “ProstateCancerData”.**

The raw data is available as an .xls EXCEL file. The file ProstateCancerData.csv contains the main table. It has clinical data for all patients (n=195), one row represents one patient data.

**Variables:**

“code of patient“ = Number to identify every patient

“Recurrent Event“= The binary variable with prostate cancer recurrence: yes=1 and no=0.

“Metastaz“= The binary variable with metastasis: yes=1 and no=0.

“Hormon therapy HT“= The binary variable with Hormone therapy: yes=1 and no=0.

“Age (year)” = Patient age (year) at starting radiotherapy

“T.Stage“= Identifies tumor stage

“Gleason score” = Identifies Gleason score

“Total.Radiation.Dose” = Total radiation dose received each patient in all fraction (Gy)

“iPSA” = Last PSA measurement pretreatment (Baseline PSA)

“lniPSA” = it’s natural logarithm of iPSA.

“slope”= The slope of linear regression on time, it was calculated for every patient. PSA repeated measurements was as dependent variable and time duration (months) between each PSA testing time and previous test time was as independent variable. Time for baseline (PSA) was assumed zero.

“follow up time (months)” = Time interval from baseline PSA test to recurrence time or end of study for patient as censored.

“number of repeated PSA” = Number of actual repeated measurement PSA after radiotherapy for each patient.

**2- “PSArepeatedmeasurements”,**

The additional file contains trajectory PSA after radiotherapy for each patient. For every patient, there are many rows that show actual repeated measurements of PSA for each patient pre- and post- radiotherapy.

**Variables:**

Row= number of PSA data

“Code patient“= Number to identify each patient

“Number of patient“= Identifies an ID number for each patient

“AG_PSA”= Actual PSA measurement for pre- or post- treatment.

“Event“= The binary variable with prostate cancer recurrence: yes=1 and no=0.

“Metastaz“= The binary variable with metastasis: yes=1 and no=0.

“HT“= The binary variable with Hormone therapy: yes=1 and no=0.

“Age” = Patient age (year) at starting radiotherapy

“T.Stage“= Identifies tumor stage

“Gleason” = Identifies Gleason score

“Radiation.Dose” = Radiation dose received each patient in fraction (Gy)

“Fraction”=Number of fraction

“Total.Radiation.Dose” = Total radiation dose received each patient in all fraction (Gy)

“iPSA” = Last PSA measurement pretreatment (Baseline PSA)

“lniPSA” = it’s natural logarithm of iPSA.

“Startday“= Time from baseline PSA measuring to repeated PSA (day).

“Follow-up Time (day)”= Time from baseline to end of study
