# Supplementary material for: Radiation dose-response (a Bayesian model) in the radiotherapy of the localized prostatic adenocarcinoma: the reliability of PSA slope changes as a response surrogate endpoint
Source: PeerJ. 2019 Jul 1;7:e7172. doi: 10.7717/peerj.7172 (PMC6610535; doi:10.7717/peerj.7172)
Supplement: Supplemental Information 5 — In this study data was analyzed based on MCMC method with using of OpenBUGS and BOA package of R for two Models. Bayesian output analysis program (boa) is available as an open-source package for the R system. The package is publicly available at http://CRAN.R-project.org/. [file peerj-07-7172-s005.docx]

**R code and links to the BOA package**

In this study data was analyzed based on MCMC method with using of OpenBUGS and BOA package of R for two Models. The code that we wrote is following.

**Appendix 1: Normal Hierarchical Model (Bellera CA. et al. 2008) code in OpenBUGS**

**model** **{**

# definition of likelihood function

for ( j in 1:Nslope) {

slope[j]~dnorm( mu[dose[j]],tau.slope )

}

# prior and hyperprior distributions

mu[1]~dnorm(mu0,tau.mu[1])

tau.mu[1]<-s[1]*tau

for (i in 2:Ndose) {

mu[i]~dnorm( mu[i-1], tau.mu[i] )

tau.mu[i]<-s[i]*tau

}

mu0~dnorm(0,1000)

tau.slope ~ dgamma( 0.001 , 0.001 ) # precision parameter for slope

sigma2.slope<-1/tau.slope

sigma<-sqrt(sigma2.slope)

tau ~ dgamma( 0.001 , 0.001 ) # precision parameter for mu

**}**

**INITS**

list( tau.slope= 0.001 , tau= 0.001 )

list( tau.slope= 0.01 , tau= 0.01 )

list( tau.slope= 0.1 , tau= 0.1 )

list( tau.slope= 1 , tau= 1 )

**DATA**

list( )

**Appendix 2: Regression Normal Model code in OpenBUGS (Leininger TJ. 2009, Ntzoufras I. 2011)**

**model {**

# definition of likelihood function

for ( i in 1:Nslope) {

slope[i]~dnorm( mu.slope[i],tau.slope )

mu.slope[i]<-alpha+beta[1]*Gleason1[i]

+beta[2]*Gleason2[i]+beta[3]*Gleason3[i]

+beta[4]*T.stage1[i]+beta[5]*T.stage2[i]

+beta[6]*HT[i]+beta[7]*iPSA[i]

+beta[8]*Age[i]

}

# calculate the posterior probability

for (j in 1:p) { p.beta[j] <- step(beta[j]) }

# prior for regression coefficients

alpha ~ dnorm(0,1000)

for (j in 1:p) { beta[j] ~ dnorm(0,1000)}

# gamma prior for precision parameter

tau.slope ~ dgamma( 0.001 , 0.001 )

# deterministic calculation of variance

sigma2.slope<-1/tau.slope

sigma<-sqrt(sigma2.slope)

# calculation of the sample variance

for (i in 1:Nslope) { c.slope[i] <- slope[i]-mean(slope[]) }

s2<- inprod( c.slope[],c.slope[] ) / (Nslope-1)

# calculation of Bayesian R squared

R2B <- 1-sigma2.slope/s2

# expected mean for slope of dose level less than 50

E.mu <- alpha+beta[1]*mean(Gleason1[])+beta[2]*mean(Gleason2[])

+beta[3]*mean(Gleason3[])+beta[4]*mean(T.stage1[])

+beta[5]*mean(T.stage2[])+beta[6]*mean(HT[])

+beta[7]*mean(iPSA[])+beta[8]*mean(Age[])

**}**

**INITS**

list(tau.slope=0.1,alpha=0,beta=c(0,0,0,0,0,0,0,0))

list(tau.slope=0.01,alpha=0,beta=c(0.1,0.1,0.1,0.1,0.1,0.1,0.1,0.1))

list(tau.slope=0.01,alpha=0,beta=c(0.01,0.01,0.01,0.01,0.01,0.01,0.01,0.01))

list(tau.slope=0.001,alpha=0.001,beta=c(0.001,0.001,0.001,0.001,0.001,0.001,0.001,0.001))

**DATA**

list( )

**Appendix 3: Package ‘boa’ (reference and links to the software)**

Bayesian output analysis program (boa) is available as an open-source package for the R system for statistical computing. The package is publicly available from the Comprehensive R Archive Network at <http://CRAN.R-project.org/>.

install.packages("boa")

library("boa")

boa.menu()

Reference:

1. Smith BJ. 2007. Boa: An R Package for MCMC Output Convergence Assessment and Posterior Inference. Journal of Statistical Software 21:1-37.
2. **Title** Bayesian Output Analysis Program (BOA) for MCMC
   **Version** 1.1.8-2
   **Date** 2016-06-22
   **Maintainer** Brian J. Smith <brian-j-smith@uiowa.edu>
   **Depends** R (>= 2.7)
   **Imports** graphics, grDevices, stats, utils
   **Description** A menu-driven program and library of functions for carrying out
   convergence diagnostics and statistical and graphical analysis of Markov
   chain Monte Carlo sampling output.
   **License** GPL-2
   **URL** http://www.jstatsoft.org/v21/i11
   **Needs Compilation** no
   **Author** Brian J. Smith [aut, cre]
   **Repository** CRAN
   **Date/Publication** 2016-06-23 01:29:05
